# Systematic Review to Gauge the Effect of Levothyroxine Substitution on Progression of Diabetic Nephropathy in Patients With Hypothyroidism and Type 2 Diabetes Mellitus

**DOI:** 10.7759/cureus.44729

**Published:** 2023-09-05

**Authors:** Prabhleen Kaur Manshahia, Shamsun Nahar, Srishti Kanda, Uzair Chatha, Victor A Odoma, Aakanksha Pitliya, Esraa M AlEdani, Japneet K Bhangu, Khalid Javed, Safeera Khan

**Affiliations:** 1 Internal Medicine, California Institute of Behavioral Neurosciences & Psychology, Fairfield, USA; 2 Medicine, All India Institute of Medical Sciences, Rishikesh, Rishikesh, IND; 3 Internal Medicine, JCMI (Jean Charles Medical Center), Orlando, USA; 4 Research, California Institute of Behavioral Neurosciences & Psychology, Fairfield, USA; 5 Cardiovascular/Oncology, IU (Indiana University) Health, Bloomington, USA; 6 Dermatology and Internal Medicine, California Institute of Behavioral Neurosciences & Psychology, Fairfield, USA; 7 Anesthesiology and Internal Medicine, California Institute of Behavioral Neurosciences & Psychology, Fairfield, USA

**Keywords:** diabetic nephropathy, hypothyroidism, levothyroxine, thyroxine (t4), type 2 diabetes mellitus (dm)

## Abstract

Diabetes mellitus (DM) and thyroid dysfunction are two disorders that are closely related. This systematic review aimed to investigate the effect of levothyroxine supplementation on diabetic nephropathy in type 2 diabetic patients with co-existing thyroid dysfunction. We explored medical databases such as PubMed, Medline, Multidisciplinary Digital Publishing Institute (MDPI), and Cochrane Library for relevant medical literature. The papers were screened, and 12 research papers involving 10,371 patients were identified after applying eligibility criteria and quality assessment using Preferred Reporting Items for Systematic Reviews and Meta-Analyses (PRISMA) guidelines. The included papers analyzed the effect of aberrant thyroid profile on kidney disease in diabetic individuals and the role that achieving euthyroid status with levothyroxine supplementation could play in diabetic nephropathy. Reduced free triiodothyronine (FT3) was the most common independent factor associated with diabetic microvascular and macrovascular complications. Levothyroxine (LT4) regimen was more effective than the placebo in lowering urinary albumin excretion rate (UAER), low-density lipoprotein cholesterol, and uric acid and decreasing oxidative stress overall. However, replacement therapy's effect may differ in the short and long terms. Thyroid hormone replacement therapy (THRT) may reduce the risk of diabetic nephropathy and cardiovascular disease (CVD) development in hypothyroid patients, but more randomized trials are needed to confirm the effect of THRT.

## Introduction and background

Diabetes mellitus (DM) affects slightly more than 500 million individuals across the globe, accounting for more than 10.5% of the adult population. This percentage is expected to rise to 12.2% (783.2 million) by 2045 [1]. Diabetes mellitus is a serious, chronic disease that arises when blood glucose levels rise due to the body's inability to secrete any or enough of the hormone insulin or to use the insulin efficiently as it does. Long-term insulin deficiency can damage many of the body's organs, resulting in disabling and grave complications that are broadly classified as macrovascular (such as cardiovascular diseases) and microvascular (nerve damage, kidney damage, and eye disease) [2]. In observational research spanning 28 nations across various continents, complications of type 2 diabetes mellitus (T2DM) are rather widespread, with 50% of diabetic patients presenting with microvascular problems and 27% with macrovascular complications [3].

Diabetic nephropathy (DN), an extremely common microvascular consequence of T2DM, is also one of the leading causes of end-stage renal disease globally. It is responsible for an increase in morbidity and death in diabetic patients. Although standard treatments such as stringent glycemic control, limiting protein intake, blood pressure control, and renin-angiotensin-aldosterone system suppression may benefit diabetic patients, they cannot entirely prevent the advancement of DN [4].

Thyroid illnesses, similar to DM, are prevalent endocrine system problems. According to ongoing research, aberrant thyroid function may influence glucose metabolism, insulin sensitivity, and the progression of long-term diabetic complications. This suggests that T2DM and its long-term complications could be linked to aberrant thyroid gland activity [5]. Hypothyroidism has been identified as a potential cause of hypoglycemia and, as such, should be monitored in cases of unexplained low blood glucose levels; on the opposite end of the spectrum, some, but not all, investigations have discovered a link between hypothyroidism and insulin resistance. Thyroid hormones, particularly free thyroxine (fT4) and free triiodothyronine (fT3), have been shown to have both agonism and antagonism to insulin in various organs [6]. Thyroid hormones have lately become a focus of diabetic kidney disease (DKD) related studies, many of which have verified that hypothyroidism or subclinical hypothyroidism has become notably more prevalent in DKD patients in recent years. The studies have also confirmed that diabetic patients with hypothyroidism have a higher risk of DKD and diabetic retinopathy [7]. In this systematic review, we aim to explore the effect of levothyroxine substitution in type 2 diabetic patients with hypothyroidism. We also aimed to explore its effect on the progression of diabetic nephropathy and the patient's overall prognosis.

## Review

Methodology

The Preferred Reporting Items for Systematic Review and Meta-Analysis (PRISMA) 2020 standards were used to perform this systematic review [8].

Search Sources and Strategy

We searched for pertinent literature in PubMed, PubMed Central (PMC), Medline, Cochrane Library, and Multidisciplinary Digital Publishing Institute (MDPI). We searched all databases employing various combinations of hypothyroidism, levothyroxine, diabetes mellitus, and diabetic nephropathy. However, when using PubMed, in addition to these keywords, the following strategy was developed and executed to search relevant medical literature in PubMed's database: (((hypothyroidism [Medical Subject Headings (MeSH) Terms]) AND (thyroxine [MeSH Terms])) AND (diabetic nephropathy [MeSH Terms])) AND (type 2 diabetes mellitus [MeSH Terms]). Table 1 shows the databases used and the identified papers per database.

**Table 1:** Keywords/search strategy used and the number of identified papers MDPI: Multidisciplinary Digital Publishing Institute; MeSH: Medical Subject Headings.

Search strategy	Database	Number of papers identified
(((hypothyroidism [MeSH Terms]) AND (thyroxine [MeSH Terms])) AND (diabetic nephropathy [MeSH Terms])) AND (type 2 diabetes mellitus [MeSH Terms])	PubMed (MeSH Strategy)	6
levothyroxine AND diabetic nephropathy	PubMed (Regular search)	32
(((hypothyroidism) AND (thyroxine)) AND (diabetic nephropathy)) AND (type 2 diabetes mellitus)	PubMed (Advanced Field search)	9
L-thyroxine and diabetic nephropathy	Medline	34
levothyroxine AND diabetic nephropathy	Cochrane Library	1
levothyroxine AND diabetes mellitus	Cochrane Library	7
hypothyroidism AND diabetic nephropathy	Cochrane Library	5
hypothyroidism AND diabetes mellitus	MDPI	28
Total number of research papers identified		122
Number of articles after removing duplicates		64

Inclusion and Exclusion Criteria

We chose literature and articles published in the last 20 years, including papers authored in English or if a full-text English-language translation was available. We included research publications involving only human subjects, with no age restrictions. If the complete text of the papers could not be retrieved, the articles were omitted. Articles addressing diabetic kidney disease in intensive care unit patients and pregnant women were also excluded. Gray literature and letters to the editor were also excluded from consideration.

Quality Assessment of the Studies

The quality of the shortlisted articles was assessed using the appropriate quality rating tools. All co-authors were involved in quality checks of the collected literature. We employed the Newcastle-Ottawa tool to assess the quality of observational studies, while the Cochrane bias assessment tool was used to assess the quality of systematic reviews. The systematic review only included studies that passed the quality appraisal.

Data Collection Process

The primary outcomes and other necessary information were assessed once the papers were finalized and extracted for the systematic review. PM and SN extracted the data independently, and all authors contributed equally to finalizing the information collected and outcomes obtained using the data extraction questionnaires.

Results

Study Identification and Selection

**Figure 1:**
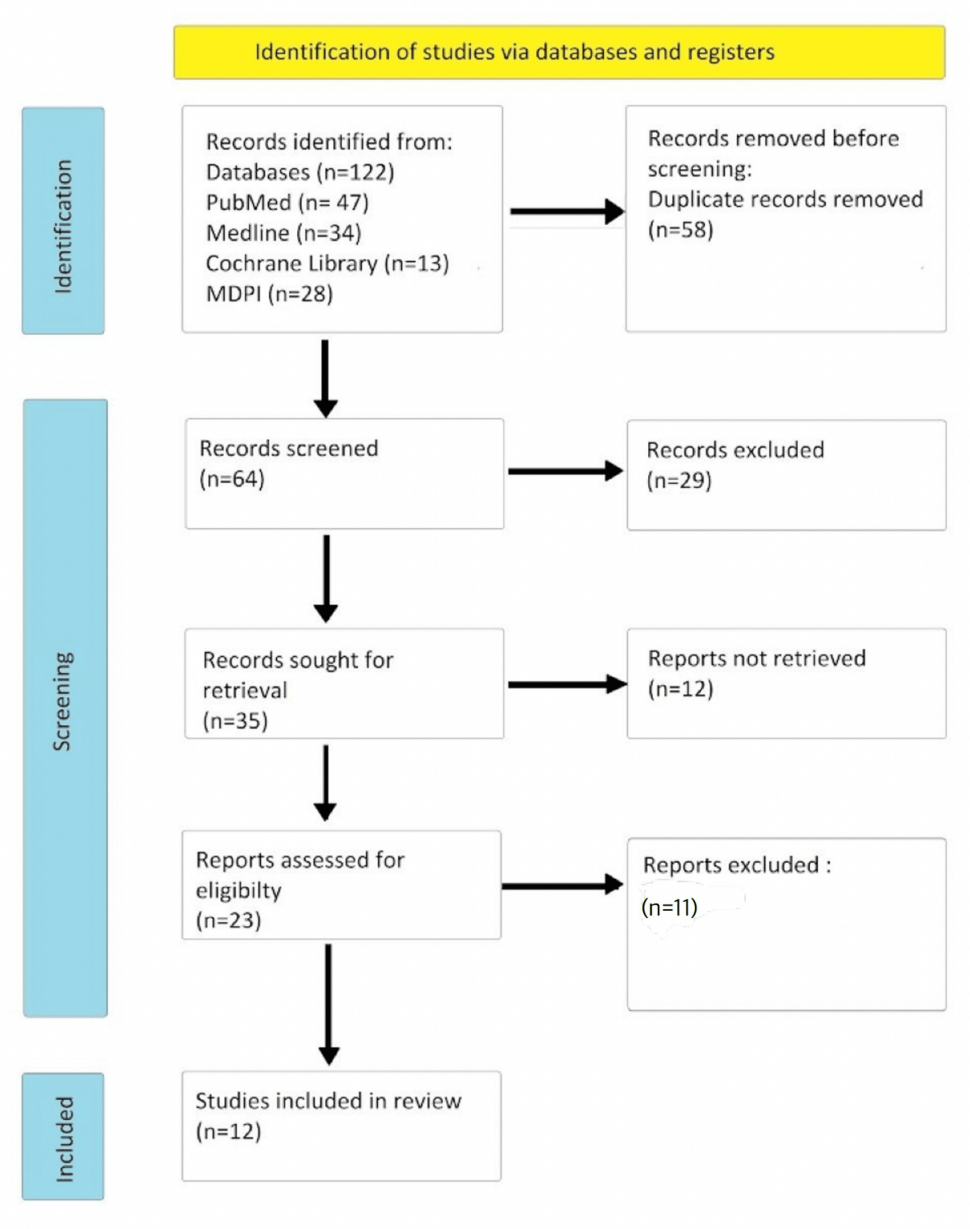
PRISMA flowchart showing the process of article selection PRISMA: Preferred Reporting Items for Systematic Reviews and Meta-Analysis; MDPI: Multidisciplinary Digital Publishing Institute.

Using all databases, our research yielded 122 relevant articles overall. A total of 58 duplicate articles were deleted before being thoroughly screened. Twenty-three papers were shortlisted after screening by reviewing titles and abstracts and retrieving complete texts. The eligibility and quality of the shortlisted full-text papers were evaluated, and 12 articles were ultimately chosen for review. Figure 1 of the PRISMA flow chart depicts the study selection process.

The articles were assessed for eligibility using the relevant quality appraisal tools. Table 2 shows the results of the quality appraisal.

**Table 2:** Quality appraisal using the Newcastle-Ottawa tool The Newcastle-Ottawa Scale quality instrument is scored by awarding a point for each answer that is marked with an asterisk (*). ** denotes comparability. *** denotes outcomes. **** denotes selection.

Name of the article	Study	Selection	Comparability	Outcome
Free triiodothyronine levels are associated with diabetic nephropathy in euthyroid patients with type 2 diabetes [4]	Wu et al., 2015	****	*	**
Evaluation of the thyroid characteristics and correlated factors in hospitalized patients with newly diagnosed type 2 diabetes [5]	Li et al., 2022	***	*	***
Association between thyroid hormones and diabetic kidney disease in Chinese adults [7]	Liu et al., 2023	***	**	***
Subclinical hypothyroidism is a risk factor for nephropathy and cardiovascular diseases in type 2 diabetic patients [9]	Chen et al., 2007	***	**	**
Effects of thyroid status on glycated hemoglobin [10]	Bhattacharjee et al., 2017	***	**	***
Thyroid function, cardiovascular events, and mortality in diabetic hemodialysis patients [11]	Drechsler et al., 2014	***	**	***
Thyroid parameters and kidney disorder in type 2 diabetes [12]	Chen et al., 2020	***	**	***
Thyroid hormones and diabetic nephropathy: An essential relationship to recognize [13]	Han et al., 2019	****	*	***
Association of thyroid hormone levels with microvascular complications in euthyroid type 2 diabetes mellitus patients [14]	Hu et al., 2022	***	*	***
Thyroid hormone replacement reduces the risk of cardiovascular diseases in diabetic nephropathy patients with subclinical hypothyroidism [15]	Seo et al., 2018	***	*	**

Outcomes Measured

The primary outcomes extracted from the finalized research papers were the effect of thyroid hormone levels, development, and progression of diabetic kidney disease. The secondary outcomes evaluated were the effect on glycated hemoglobin levels and the overall effect on microvascular and macrovascular complications of type 2 diabetes mellitus.

Study Characteristics

We reviewed 12 research papers involving 10,371 total patients. Two finalized studies were randomized clinical trials, with the other 10 being observational studies. All studies involved type 2 diabetic patients and the measurement of thyroid hormones and their role in the development and progression of diabetic nephropathy. Two of the studies focused on the effect of levothyroxine therapy on diabetic kidney disease. Renal failure and dialysis or other outcomes, such as other microvascular and macrovascular complications of diabetes mellitus, were not documented in all studies. Few studies compared glycated hemoglobin levels in patients with thyroid abnormalities and assessed if such changes in glycated hemoglobin or hemoglobin A1c (HbA1c) can be reversed upon achieving euthyroid status. Studies that compared the group that achieved euthyroid status with the group not on levothyroxine therapy showed relatively reduced renal damage. Table 3 shows a summary and characteristics of all involved papers.

**Table 3:** Summary of the included studies DN: diabetic nephropathy; FT3: free triiodothyronine; UACR: urine albumin-to-creatinine ratio; FT4: free thyroxine; T3: triiodothyronine; TgAb: thyroglobulin; TSH: thyroid-stimulating hormone; eGFR: estimated glomerular filtration rate; SPINA-GD: sum activity of peripheral deiodinases; TSHI: thyroid-stimulating hormone index; TTSI: thyrotroph thyroid hormone sensitivity index; UAER: urinary albumin excretion rate; LT4: levothyroxine;  SCH: subclinical hypothyroidism; TPO-Ab: anti-thyroid peroxidase antibody; MDA: malondialdehyde; 8-OHdG: 8-hydroxyguanosine; mAlb: microalbumin; SOD: superoxide dismutase, HbA1c: hemoglobin A1c; T2DM: type 2 diabetes mellitus.

Name of the article	Authors and year of publication	Type of the study	Purpose of the study	Number of participants	Results	Conclusions
Free triiodothyronine levels are associated with diabetic nephropathy in euthyroid patients with type 2 diabetes [4]	Wu et al., 2015	Cross-sectional study	To examine the relationship between thyroid activity and diabetic nephropathy (DN) in euthyroid type 2 diabetes patients.	421	Among the 421 patients, 203 (48.2%) had DN and had considerably lower FT3 levels than those who did not have DN. FT3 levels were positively related to the estimated glomerular filtration rate and inversely linked to UACR.	In euthyroid patients with type 2 diabetes, serum FT3 levels are inversely related to DN, independent of established risk factors.
Evaluation of the thyroid characteristics and correlated factors in hospitalized patients with newly diagnosed type 2 diabetes [5]	Li et al., 2022	Observational study	To study thyroid hormone levels, diabetic complications, and metabolic parameters in hospitalized patients with recently diagnosed type 2 diabetes.	340	The levels of free triiodothyronine, free thyroxine, and thyroid-stimulating hormone were considerably lower in diabetic patients compared to the non-diabetic control group. Thyroid problems were found in 21.2% of diabetic patients, compared to 4.2% of controls. The amount of FT3 was inversely associated with urine total protein (mg/24 h) and the presence of DN.	Thyroid hormone levels are linked to diabetic complications and diabetes-related metabolic and demographic variables. Reduced FT3 is closely linked to the presence of DN.
Association between thyroid hormones and diabetic kidney disease in Chinese adults [7]	Liu et al., 2023	Case-control study	To investigate the relationship between thyroid hormones and various stages of diabetic nephropathy in Chinese adults.	2,832	Following propensity score matching on age, gender, hypertension, HbA1c, total cholesterol, serum triglyceride, and diabetes duration, each 0.2 pg/mL increase in serum-free triiodothyronine (FT3) was associated with a 13%, 22%, and 37% decreased risk of moderate-risk, high-risk, and very-high-risk diabetic nephropathy stages, respectively, relative to low-risk nephropathy.	High serum FT3 concentrations were related to a significantly lower risk of moderate- to very-high-risk diabetic nephropathy.
Subclinical hypothyroidism is a risk factor for nephropathy and cardiovascular diseases in type 2 diabetic patients [9]	Chen et al., 2007	Cross-sectional study	To investigate the association between subclinical hypothyroidism and the prevalence of retinopathy and nephropathy, cardiovascular disease, and death in type 2 diabetic individuals not on thyroxine replacement therapy.	588	After adjusting for age, gender, HbA1C, other standard cardiovascular risk factors, and medication, the risk of cardiovascular events was significantly increased in people with type 2 diabetes with subclinical hypothyroidism. Still, it became nonsignificant after adjusting for the urine albumin-to-creatinine ratio.	Patients with type 2 diabetes with subclinical hypothyroidism have an elevated risk of nephropathy and cardiovascular events but not retinopathy.
Effects of thyroid status on glycated hemoglobin [10]	Bhattacharjee et al., 2017	Case-control study	To investigate the effects of altered thyroid function on HbA1c levels in people without diabetes, with overt hyper- and hypothyroidism, and if such changes in HbA1c can be reversed upon achieving euthyroid status.	127	Following treatment, there was a significant decrease in HbA1c and an increase in reticulocyte count in the hypothyroid group, but there was no change in glucose level. In the hyperthyroid group, HbA1c was not affected significantly after treatment.	HbA1c levels were notably higher in hypothyroid patients at baseline but decreased considerably after attaining euthyroidism with no change in glucose levels.
Thyroid function, cardiovascular events, and mortality in diabetic hemodialysis patients [11]	Drechsler et al., 2014	Prospective cohort Study	To determine whether subclinical thyroid abnormalities were linked with cardiovascular events and mortality in diabetic hemodialysis patients.	1,000	Patients with euthyroid sick syndrome had a nearly threefold increase in short-term mortality, but no effect was evident in the long run.	The euthyroid sick syndrome is substantially linked to death in hemodialysis patients. Thyroid function should be checked regularly to help predict cardiac risk in dialysis patients.
Thyroid parameters and kidney disorder in type 2 diabetes [12]	Chen et al., 2020	Cross-sectional study	To investigate the relationship between thyroid profile and renal diseases, particularly in euthyroid diabetic subjects.	4,136	UACR (urine albumin-to-creatinine ratio) levels were shown to be inversely related to FT3 and T3. Furthermore, eGFR was positively linked with FT3 and T3 and negatively associated with TSH, FT4, and TgAb positivity.	Elevated TSH and FT4, lower FT3, and TgAb positivity were linked with greater UACR and lower eGFR levels in type 2 diabetic patients. TSH and FT4 levels and thyroid homeostasis parameters (SPINA-GD, TSHI, and TTSI) were linked to the prevalence of renal diseases.
Thyroid hormones and diabetic nephropathy: an essential relationship to recognize [13]	Han et al., 2019	Cross-sectional study	To look at the link between thyroid hormones and clinicopathologic changes in patients with biopsy-proven diabetic nephropathy.	146	Overt proteinuria (>5 g/24 h) and the degree of glomerular lesions differed significantly between the euthyroid and high-TSH groups. According to logistic regression studies, the relationships between high TSH, low FT3, and renal clinicopathologic changes were reportedly significant.	Patients with diabetic nephropathy with high TSH and/or low FT3 exhibited more severe proteinuria, renal insufficiency, and glomerular lesions, implying that thyroid hormone regulation may protect the kidneys.
Association of thyroid hormone levels with microvascular complications in euthyroid type 2 diabetes mellitus patients [14]	Hu et al., 2022	Observational study	To look at the prevalence of microvascular complications and the relationship between thyroid hormones and these complications in euthyroid type 2 diabetes mellitus patients.	248	Prevalence of a microangiopathy was found to be 72.18% (n = 179). Simultaneously, the prevalence of diabetic nephropathy (DN) was 31.85% (n=79). The odds ratio for free triiodothyronine (FT3) developing DN was 0.310. Furthermore, the odds ratio for free thyroxine (FT4) developing DN was 0.726.	The study shows that serum FT3 and FT4 levels, independent of established risk variables, are negatively linked with microangiopathy in euthyroid patients with T2DM.
Thyroid hormone replacement reduces the risk of cardiovascular diseases in diabetic nephropathy patients with subclinical hypothyroidism [15]	Seo et al. 2018	Retrospective cohort study	To see if replacing thyroxine would decrease the risk of cardiovascular disease among patients with subclinical hypothyroidism and diabetic nephropathy.	257	The prevalence of acute coronary syndrome and cerebrovascular incidents was lower in the levothyroxine replacement therapy group than in the non-treatment group. Still, there was no difference in peripheral artery disorders.	In diabetic nephropathy patients with subclinical hypothyroidism, levothyroxine replacement therapy may reduce the risk of cardiovascular disease.
Can levothyroxine treatment reduce urinary albumin excretion rate in patients with early type 2 diabetic nephropathy and subclinical hypothyroidism? A randomized double-blind and placebo-controlled study [16]	Liu et al., 2015	Randomized control trial	To explore how levothyroxine therapy affects urinary albumin excretion rate (UAER) in patients with early type 2 diabetic nephropathy and subclinical hypothyroidism who had mildly elevated thyroid-stimulating hormone levels and serum thyroid peroxidase antibody positivity.	136	Comparison of therapy-related differences between groups revealed that the LT4 regimen was more effective than the placebo in lowering UAER, low-density lipoprotein cholesterol, and uric acid.	In early type 2 DN and SCH patients with modestly elevated TSH levels and serum TPO-Ab positivity, LT4 therapy may reduce UAER and provide renal protective effects.
The effect of L-thyroxine substitution on oxidative stress in early-stage diabetic nephropathy patients with subclinical hypothyroidism: a randomized double-blind and placebo-controlled study [17]	Chen et al., 2018	Randomized control trial	To investigate the oxidative stress status of patients with early diabetic nephropathy and subclinical hypothyroidism and the influence of L-thyroxine therapy on oxidative stress in these individuals.	140	UAER, MDA, and 8-OHdG levels were greater, but SOD activity was reduced in DN patients with SCH compared to DN patients. UAER, MDA, and 8-OHdG levels in the LT4 group declined dramatically to levels no longer distinguishable from the euthyroid group. SOD activity rose noticeably. However, after 24 weeks of follow-up, the levels of mAlb, MDA, and 8-OHdG in the SCH group were higher than in the euthyroid group. SOD activity in the SCH group dropped considerably.	Oxidative stress is higher in DN patients with SCH, and SCH may aggravate renal damage in patients with early DN, and LT4 therapy may reduce oxidative stress and renal damage.

Discussion

Researchers have found that subclinical hypothyroidism (SCH) or hypothyroidism is the most common form of thyroid disease [5]. According to epidemiological research, the prevalence of SCH in T2DM patients exceeds 10% [16]. Low levels of thyroid hormones have been associated with DN in euthyroid patients with T2DM. The connection between DN and thyroid hormones has become concerning in recent years [5]. Thyroid hormones influence renal development, glomerular and tubular function, and renin-angiotensin-aldosterone system activation. Apart from directly acting on the kidney, thyroid hormones may additionally influence renal functions via cardiovascular and systemic hemodynamics. The kidney, meanwhile, regulates thyroid hormone metabolism and excretion by facilitating iodine removal by glomerular filtration. Raised levels of inorganic iodide and thyroid iodine in the serum of patients with kidney disease can prolong the Wolff-Chaikoff effect leading to hypothyroidism. Furthermore, the protein-bound thyroxine can be lost in the urine of diabetic kidney disease (DKD) patients [7].

Metabolic Parameters in Diabetic Patients

Serum thyroid hormone concentrations were reportedly related to multiple metabolic and demographic parameters. Insulin resistance is a significant factor in the link between thyroid disease (TD) and T2DM. According to a study, decreased triiodothyronine (T3) levels strongly correlated with lower homeostatic model assessment for insulin resistance (HOMA-IR) even in non-diabetic subjects, indicating an association of insulin resistance with thyroid function. Another study in euthyroid overweight people found that higher free triiodothyronine (FT3) levels were related to an increased risk of insulin resistance. Furthermore, FT3 and free thyroxine (FT4) positively and negatively correlated with HOMA-IR and deranged lipid levels. Serum FT4 was inversely linked to insulin resistance in euthyroid patients and positively linked to a risk of metabolic syndrome. Thyroid-stimulating hormone (TSH), however, was found to be positively related to insulin resistance. TSH and thyroid hormones correlate with metabolic parameters such as HOMA-IR, homeostasis model assessment of β-cell function (HOMA-β), glycated hemoglobin (HbA1c), serum C-peptide, and high-density lipoprotein-C (HDL-C) levels. Obesity was another risk factor for TD. In obese euthyroid patients, FT3 and FT4 levels were positively linked with body mass index (BMI). A large population-based study showed that raised TSH within the normal range was a risk factor for a variety of cardiometabolic changes, including central obesity, insulin resistance, dyslipidemia, high blood pressure, hyperuricemia, inflammation, and hypercoagulability [5]. In another study, while having comparable glycemic index status, the median baseline HbA1c level in overt hypothyroid cases was considerably greater than that in the matched control population. Following thyroid hormone supplementation, the median HbA1c value reduced considerably, presumably due to red blood cell (RBC) turnover in hypothyroid patients. After thyroid hormone replacement, the reticulocyte count rose. Although there was a difference in HbA1c levels in hypothyroid patients before and after attaining euthyroidism, this difference was no more of statistical significance when adjusted for retic count, implying altered HbA1c levels in hypothyroid individuals. No such change in HbA1c was noted in hyperthyroid cases. Despite a considerable change in reticulocyte count, the median HbA1c value remained unchanged following hyperthyroidism treatment [10]. 

Thyroid Hormones and Clinicopathologic Kidney Changes in Diabetic Patients

An epidemiological analysis also found that those with chronic renal disease have a higher prevalence of subclinical and clinical hypothyroidism. Along with increased urine albumin excretion rate and decreased creatinine clearance, the glomerular filtration rate in hypothyroid people is roughly one-third lower than in euthyroid people [9]. The precise mechanisms are not fully understood, but thyroid hormones play a vital part in the kidneys' growth, development, and physiology and in the maintenance of endothelial and vascular functions. T3 has been reported to increase phosphatidylinositol 3-kinase (PI3K), decrease transforming growth factor β1 (TGF-1) expression, improve structurally damaged kidneys, and lower albuminuria [5]. Furthermore, being strong glucose regulators, thyroid hormones can play a role in diabetes progression by boosting the expression of Goto-Kakizaki (GK) and V-Maf avian musculoaponeurotic fibrosarcoma oncogene homolog A (Mafa) in the pancreas, enabling the rapid maturation and renewal of β-cells, and increasing secretion of insulin in the pancreas, all of which could impact DKD development [7]. In addition, thyroid status abnormalities, particularly reduced FT3 levels and/or raised TSH levels, are associated with worse endothelial function in patients with advanced chronic kidney disease or end-stage renal disease [5]. FT3, in particular, may be associated with DKD via various mechanisms. Inflammatory cytokines such as tumor necrosis factor- and interleukin-1 can hinder the expression of type one 5'-deiodinase and thus reduce T4-to-T3 transformation [7]. The prevalence of kidney disorders in T2DM patients is inversely related to FT3 level. In T2DM patients, DN is reportedly a risk factor for TD, and they have elevated TSH levels and reduced FT3 levels, which were linked to more severe proteinuria, renal insufficiency, and glomerular lesions in DN patients. FT3 and FT4 were also found to be closely linked with estimated glomerular filtration rate (eGFR) levels.

Patients with DN had lower FT3 levels and a lower FT3/FT4 ratio [5]. High TSH and FT4 (or T4), lower FT3 (or T3), and thyroglobulin antibodies (TgAb) positivity correlated with higher urine albumin-to-creatinine ratio (UACR) and lower eGFR levels in type 2 diabetes patients. TSH and FT4 (or T4) levels and thyroid homeostasis parameters like sum activity of peripheral deiodinases (SPINA-GD), thyroid-stimulating hormone index (TSHI), and thyrotroph thyroid hormone sensitivity index (TTSI) were linked to the prevalence of kidney disorders. Even in the normal reference range, lower FT3 was most related to decreased eGFR compared to all other thyroid hormones [12]. Low FT3 levels were an independent risk factor for the onset and progression of DN in T2DM patients. Another study in adult euthyroid type 1 diabetes mellitus (T1DM) patients found that higher FT3 levels were associated with a decreased microangiopathy prevalence [5]. Previous research also indicated that a raised level of TSH in the blood is an independent risk factor for albuminuria in type 2 diabetics with subclinical hypothyroidism [17]. Hyperglycemia, an established pathogenetic factor of chronic diabetic complications, produces more reactive oxygen species and suppresses antioxidative mechanisms via glycation of scavenging enzymes, resulting in oxidative stress. Oxidative stress has been proven to increase transforming growth factor 1 (TGF-1) and fibronectin expression, two genes associated with diabetic glomerular injury.

Moreover, halting oxidative stress could alleviate all DN-associated symptoms. Han et al. [13] discovered that DN patients with high TSH and/or low FT3 had more severe proteinuria, renal insufficiency, and glomerular lesions in a study of 146 biopsy-proven T2DM-associated DN patients. These findings pointed out that regulating thyroid hormones could have a reno-protective effect. Thyroid hormone can reportedly boost the promoter activity of the renin gene in Calu-6 cells, affecting the renin-angiotensin-aldosterone system and hemodynamics and influencing the kidneys' weight, size, and structure. Hypothyroidism can also inhibit the expression of vascular endothelial growth factor and insulin-like growth factor-1, leading to glomerular basement membrane thickening and mesangial matrix expansion [13].

Influence of Levothyroxine Therapy on Diabetic Kidney Disease

Thyroid hormones regulate basal metabolic rate in normal physiological states via changes in mitochondrial oxygen consumption, the body's primary process of free radical production. Thyroid hormones have also been shown to influence antioxidant protein biosynthesis and breakdown. Malondialdehyde (MDA) and urine 8-hydroxyguanosine (8-OHdG), indicators of lipid peroxidation and oxidative tissue damage, were higher in DN patients with SCH compared to euthyroid diabetics. Superoxide dismutase (SOD) activity, a marker of antioxidant defense status, was lower, demonstrating accelerated kidney injury in hypothyroid diabetics [17]. Thyroid hormones have a significant impact on vascular and endothelial functioning. Endothelial dysfunction in SCH patients is characterized by reduced availability of nitric oxide (NO), which can be rectified with levothyroxine supplementation. Serum FT3 levels are inversely related to DN in euthyroid diabetics, regardless of other risk factors [4]. In a clinical trial by Liu et al. [16], levothyroxine (LT4) treatment was more successful than the placebo in lowering blood uric acid levels and decreasing the urinary albumin excretion rate (UAER). LT4 replacement showed a slightly upward trend in glomerular filtration rate (GFR) because it may reduce DN risk factors such as blood lipids and uric acid, hence ameliorating renal ischemia by increasing GFR [16]. Another clinical trial found that the UAER decreased and antioxidant levels increased significantly to the point where they were no longer distinguishable from the euthyroid group, implying that LT4 helps prevent glomerular injury in DN patients with SCH. Simultaneously, following LT4 replacement, decreased albuminuria was observed in DN patients with SCH, implying that LT4 therapy could benefit DN patients by reducing oxidative stress [17]. Chen et al.'s [9] observational study fails to establish that treating subclinical hypothyroidism reduces the risk of DN. de Hollander et al., on the other hand, demonstrated that thyroxine treatment improved kidney function in 32 hypothyroid individuals [18]. In one study, levothyroxine replacement reverted serum creatinine levels to baseline in three patients with hypothyroidism and renal dysfunction after several months. Another cohort study discovered that thyroid hormone replacement therapy preserved renal function and was an independent predictor of renal outcome in chronic kidney disease (CKD) patients with SCH [13].

Effect of Thyroid Abnormalities on Microvascular Complications

Other diabetic microvascular complications have been linked to thyroid disorders in diabetes. Cross-sectional studies revealed that subclinical hypothyroidism was common in diabetic retinopathy (DR) and diabetic peripheral neuropathy (DPN) patients and was strongly connected to their severity. Recent studies demonstrate that low FT3, even in the normal range, is independently associated with DR and DPN in euthyroid diabetic patients. However, in some studies, the relationships between thyroid hormone levels and diabetic microvascular complications such as DR were found to be weak or negative, and there is a lack of prospective clinical studies on how the thyroid profile affects diabetes and its complications in the long term. Chen et al. [9] observed no positive relationships between DR and aberrant thyroid profile. However, the incidence of other diabetic microvascular complications may have been underestimated because fundus examinations were only performed on patients with the corresponding symptoms. Following adjustments for typical confounding factors such as age, gender, duration of T2DM, BMI, elevated blood pressure, HbA1c, and lipid profile, the results of the study revealed that normal-low levels of free FT3 and FT4 were associated with an increased risk of microvascular complications. TSH levels, however, were only linked with DN [9]. A meta-analysis stated that subclinical hypothyroidism exacerbated the risk of DR in diabetics by 2.13 times.

Furthermore, Seo et al. [15] discovered that FT3 levels in the normal range were inversely associated with DR in euthyroid diabetic patients [14]. Wu et al. (2018) discovered, like a few previous studies, that thyroid activity has no relation to DR, implying that the protective effect of FT3 in diabetics could be specific to every organ [4]. Compared to diabetic microvascular and acute complications, evidence of a link between thyroid hormone and diabetic macrovascular complications is scarcer and more inconsistent [5].

Effect of Thyroid Abnormalities on Macrovascular Complications

Epidemiological research has suggested that subclinical hypothyroidism is a distinct risk factor for cardiovascular disease. However, whether the treatment of subclinical hypothyroidism influences cardiovascular outcomes is still unclear. According to Chu and Crapo [19], most patients with subclinical hypothyroidism appear to not benefit from thyroxine substitution, except in certain clinical situations in some individuals. Compared to non-diabetics, diabetic patients have a two to six times higher risk of dying from cardiovascular problems. Subclinical hypothyroidism increases the risk of cardiovascular events in diabetics, according to Chen et al. [9]. They concluded that levothyroxine therapy in type 2 diabetes patients is appropriate. Hypothyroidism reduces cardiac output, increases peripheral vascular resistance, and causes intrarenal vasoconstriction [9]. The effect of thyroid disease on cardiovascular mortality was investigated in a prospective follow-up study of 1,000 well-characterized diabetic hemodialysis patients. A marginal effect on the risk of sudden cardiac death was discovered in patients with subclinical hyperthyroidism. Patients with euthyroid sick syndrome had a significantly higher mortality rate in the first year, which then decreased. But there were no significant findings in patients with subclinical hypothyroidism, possibly because the cohort was rather small to conclude [11]. High TSH levels have been linked to an increased incidence or risk of cardiovascular disease, particularly in obese patients. In a cross-sectional study, total thyroid hormones were not found to be independent risk factors for cardiovascular events in patients with T2DM. Another study in euthyroid patients with T2DM found that low but clinically normal free thyroid hormone levels were linked to an increased risk of diabetic macrovascular complications. Li et al. (2022) discovered that as FT3 and FT4 levels decreased, the prevalence of carotid atherosclerosis increased [5]. Even after controlling for baseline serum TSH levels, thyroxine replacement correlated with a lower incidence of cardiovascular disease (CVD). The primary contributors to increased CVD risk in diabetics are endothelial dysfunction, systemic inflammation, dyslipidemia, hypercoagulability, and cardiac autonomic neuropathy [15]. Another observational study also demonstrated that low-normal FT3 and FT4 levels are associated with an increased risk of macrovascular complications. Hence, appropriate management with oral L-T4 monotherapy is crucial [14].

Limitations

The studies selected were mostly observational, with a wide spectrum of sample sizes and follow-up periods. Not every research study analyzed the same variables and outcomes. So far, very few clinical trials have been done. Our systematic review excludes research papers written in any language other than English. Excluding research papers published in other languages can omit important information and relevant data.

## Conclusions

This systematic review was conducted to investigate the effect of levothyroxine replacement therapy on the progression of diabetic nephropathy in diabetic patients with co-existing hypothyroidism and to assess if starting levothyroxine in such patients had any other systemic effects of decreasing other comorbidities. Based on the articles reviewed, levothyroxine reduces oxidative stress, decreases UAER, and provides an overall reno-protective effect in diabetic patients. Patients with type 2 diabetes with subclinical hypothyroidism have an elevated risk of nephropathy and cardiovascular events but not retinopathy. The incidence of CVD was frequent in patients with T2DM with albuminuria and SCH.

Thyroid function should be checked regularly to help predict cardiac risk in dialysis patients. Early checkups for thyroid dysfunction can help improve the overall prognosis of patients by assisting the management approach in end-stage diabetic kidney disease patients on dialysis by predicting the risk of cardiovascular morbidity. Decreased FT3 is the most associated independent factor in diabetic microvascular and macrovascular complications. However, because studies were not for the same duration, the effect of replacement therapy may vary in the short and long terms. The mechanism of LT4 therapy for protecting kidney function requires further research. Levothyroxine replacement therapy may lower the risk of diabetic nephropathy and CVD development in hypothyroid patients, but more randomized trials confirming the effect of levothyroxine therapy are needed.
