# Correction: Systematic Review to Gauge the Effect of Levothyroxine Substitution on Progression of Diabetic Nephropathy in Patients With Hypothyroidism and Type 2 Diabetes Mellitus

**DOI:** 10.7759/cureus.c306

**Published:** 2025-09-23

**Authors:** Prabhleen Kaur Manshahia, Shamsun Nahar, Srishti Kanda, Uzair Chatha, Victor A Odoma, Aakanksha Pitliya, Esraa M AlEdani, Japneet K Bhangu, Khalid Javed, Safeera Khan

**Affiliations:** 1 Internal Medicine, California Institute of Behavioral Neurosciences & Psychology, Fairfield, USA; 2 Medicine, All India Institute of Medical Sciences, Rishikesh, Rishikesh, IND; 3 Internal Medicine, JCMI (Jean Charles Medical Center), Orlando, USA; 4 Research, California Institute of Behavioral Neurosciences & Psychology, Fairfield, USA; 5 Cardiovascular/Oncology, IU (Indiana University) Health, Bloomington, USA; 6 Dermatology and Internal Medicine, California Institute of Behavioral Neurosciences & Psychology, Fairfield, USA; 7 Anesthesiology and Internal Medicine, California Institute of Behavioral Neurosciences & Psychology, Fairfield, USA

This article has been corrected to fix the following typo in the PRISMA diagram: Reports excluded corrected from 9 to 11.

